# Maternal serum sex hormone–binding globulin and androgen levels in fetuses with intrauterine growth restriction: a prospective case–control study

**DOI:** 10.1007/s00404-026-08440-0

**Published:** 2026-05-12

**Authors:** Mediha Kübra Ceylan, Ömer Faruk Öz, Benan Kahraman Ersoy, Ayşe Yavuz, Fatma Atmaca

**Affiliations:** 1 Department of Obstetrics and Gynecology, Istanbul Training and Research Hospital, University of Health Sciences, Istanbul, Turkey; 2 Department of Obstetrics and Gynecology, Faculty of Medicine, Akdeniz University, Antalya, Turkey

**Keywords:** Fetal growth restriction (FGR), Sex hormone-binding globulin (SHBG), Free androgen index (FAI), Maternal androgens, Placental dysfunction

## Abstract

**Purpose:**

To compare maternal serum sex hormone-binding globulin (SHBG) and androgen markers in intrauterine growth restriction (IUGR) and gestational-age-matched controls, and to assess their associations with fetal growth.

**Methods:**

This prospective case–control study included 45 IUGR pregnancies and 50 controls. Blood was collected in the late third trimester, preferentially antepartum; otherwise within 24 h postpartum (IUGR: 36/45 antepartum, 9/45 postpartum; controls: 9/50 antepartum, 41/50 postpartum). Total testosterone, dehydroepiandrosterone sulfate, estradiol, androstenedione, and SHBG were measured by immunoassay. FAI was calculated as (testosterone [nmol/L]/SHBG [nmol/L]) × 100 after conversion of testosterone from ng/mL to nmol/L. Sensitivity analyses compared hormone levels within antepartum and postpartum subgroups. Exploratory multivariable linear regression in the IUGR group assessed whether SHBG and total testosterone were independently associated with birth weight percentile and abdominal circumference percentile after adjustment for maternal age, systolic blood pressure, and sampling time.

**Results:**

IUGR mothers were older, delivered earlier, and had higher systolic blood pressure than controls. Birth weight was lower in IUGR (2190 ± 624 g vs 3306 ± 391 g). SHBG was lower in IUGR (257 ± 117 vs 382 ± 106 nmol/L, *p* < 0.001), whereas FAI was modestly higher (1.27 ± 1.06 vs 0.85 ± 0.71, *p* = 0.018). In the antepartum-only subgroup, SHBG remained lower in IUGR, whereas FAI did not differ significantly. Unadjusted correlations of SHBG and total testosterone with fetal growth parameters in IUGR were attenuated after adjustment.

**Conclusion:**

IUGR was associated with lower maternal SHBG. The FAI difference was modest and not robust in the antepartum-only analysis. Hormone-growth associations were exploratory and were not independently significant after adjustment.

## What does this study add to clinical work?

**Table:** 

Pregnancies complicated by intrauterine growth restriction showed significantly lower maternal SHBG levels than gestational age-matched controls, whereas the apparent increase in free androgen index was modest and not robust in the antepartum-only analysis. These findings suggest that altered maternal sex hormone binding may accompany IUGR in late pregnancy, although the clinical utility of these markers requires confirmation in larger prospective studies.	

## Introduction

Intrauterine growth restriction (IUGR), also referred to as fetal growth restriction (FGR), describes a fetus that fails to reach its genetic growth potential in utero [1, 2]. It complicates an estimated 5–10% of pregnancies and remains a major cause of perinatal morbidity and mortality worldwide [2]. IUGR is associated with increased risks of stillbirth, neonatal complications, and long-term health consequences for the child, including higher susceptibility to metabolic and cardiovascular diseases later in life [3, 4]. The etiology of IUGR is multifactorial, but placental insufficiency is a central underlying mechanism in most cases [2]. Suboptimal placental perfusion and function lead to chronic fetal nutrient and oxygen deprivation, resulting in diminished fetal growth.

During normal pregnancy, the maternal endocrine environment undergoes profound changes to support fetal development. In particular, circulating estrogen levels rise substantially as the placenta produces large amounts of estriol and estradiol; this estrogen surge stimulates hepatic production of sex hormone-binding globulin (SHBG), leading to a several-fold increase in maternal SHBG concentrations by the early second trimester[5]. SHBG binds testosterone and other androgens with high affinity, thus regulating the bioavailable (free) fraction of androgens. Maternal androgens also change throughout gestation: for instance, testosterone levels increase in late pregnancy as part of the normal endocrine adaptation [5]. Excessively high maternal androgen levels, however, can have deleterious effects on the pregnancy [6]. Prenatal exposure to elevated androgens has been linked to reduced birth weights and impaired fetal growth, possibly through direct and indirect disruption of placental function [7, 8]. Preclinical studies and clinical observations have indicated that maternal hyperandrogenemia may contribute to uteroplacental insufficiency. In animal models, testosterone administration can reduce placental and fetal weights and increase placental vascular resistance [8]. In humans, conditions characterized by hyperandrogenism, such as polycystic ovary syndrome (PCOS), are associated with higher rates of pregnancy complications including preeclampsia, fetal growth restriction, and small-for-gestational-age infants [9, 10]. A recent cohort study demonstrated that pregnant women with PCOS (who tend to have elevated free androgen levels and lower SHBG) delivered infants with significantly lower mean birth weight than unaffected women, even after adjusting for confounders [7]. Maternal SHBG levels are generally lower in pregnancies affected by PCOS, reflecting an environment of increased circulating free androgens [10–12].

Despite these indications, the specific relationship between maternal androgenic profile and idiopathic IUGR (in the absence of overt maternal endocrine disorders) remains incompletely understood. Prior studies on maternal hormones in growth-restricted pregnancies have yielded limited and sometimes inconsistent results. Historically, low maternal estriol (an estrogen produced by the placenta) was noted in pregnancies with severe IUGR, and urinary estriol excretion was once used as a marker of placental function [13, 14]. More recent research supports that maternal estradiol levels tend to be lower at delivery in IUGR pregnancies compared to normal pregnancies, and that estradiol concentrations correlate positively with placental efficiency [15, 16]. Salas et al. reported that by ~ 34 weeks’ gestation, women who would go on to deliver small-for-gestational-age babies had significantly reduced serum estradiol and progesterone levels compared to those with normal fetal growth [17]. However, other investigators found that first- and second-trimester maternal SHBG or androgen levels did not predict later IUGR [18, 19], suggesting that endocrine differences may become evident only in the late stage of pregnancy when placental insufficiency is pronounced. Overall, there is a paucity of data focusing on maternal free and bound androgen levels in established IUGR cases.

Given this background, we hypothesized that pregnancies complicated by IUGR would exhibit alterations in maternal SHBG and androgen levels relative to normal pregnancies, reflecting the hormonal impact of placental dysfunction. In this study, we aimed to compare maternal serum SHBG, total testosterone, androstenedione, dehydroepiandrosterone sulfate (DHEA-S), estradiol, and calculated free androgen index between IUGR and non-IUGR pregnancies near term. We also explored whether these hormonal parameters correlate with the degree of fetal growth restriction, as assessed by fetal biometric percentiles, to gain insight into potential pathophysiological links and to evaluate their utility as biochemical markers for IUGR.

## Materials and methods

### Study design and participants

This prospective case–control study was conducted at the Department of Obstetrics and Gynecology, University of Health Sciences, Istanbul Education and Research Hospital (Istanbul, Turkey). The protocol was approved by the institutional ethics committee (04 June 2021; decision No. 2861), and written informed consent was obtained from all participants. Third-trimester singleton pregnancies with fetal growth restriction (FGR) and gestational-age-matched controls with appropriate fetal growth were enrolled.

FGR was defined as estimated fetal weight (EFW) or abdominal circumference (AC) at or below the 10th percentile for gestational age, based on the INTERGROWTH-21st standards. Gestational age was confirmed by first-trimester crown-rump length. Controls had both EFW and AC above the 10th percentile; controls were frequency-matched to cases by gestational age at inclusion.

Exclusion criteria for both groups included hypertensive disorders of pregnancy (including pre-eclampsia), pre-gestational diabetes or gestational diabetes requiring medication, maternal endocrine disorders affecting hormone levels (e.g., polycystic ovary syndrome or adrenal disease), fetal aneuploidy or major congenital anomalies, intrauterine fetal demise, and multiple gestations.

### Data collection and hormone measurements

Maternal demographics and obstetric variables (age, gravidity, parity, body mass index), blood pressure, oligohydramnios (amniotic fluid index < 5 cm or deepest vertical pocket < 2 cm), fetal biometry, and EFW percentiles were recorded at enrollment; delivery outcomes were abstracted from records.

Maternal venous blood (approximately 16 mL) was obtained in the late third trimester, preferentially antepartum; when antepartum sampling was not feasible, samples were collected within 24 h postpartum. Sampling distribution was IUGR: 36/45 (80.0%) antepartum and 9/45 (20.0%) postpartum; controls: 9/50 (18.0%) antepartum and 41/50 (82.0%) postpartum. Time of day and fasting status were not standardized or routinely recorded. Samples were processed in the institutional laboratory, and sera were stored at 4 °C until assay.

Total testosterone, estradiol, dehydroepiandrosterone sulfate (DHEA-S), and sex hormone-binding globulin (SHBG) were measured by automated electrochemiluminescence immunoassay (Roche Cobas 8000, Roche Diagnostics, Germany). Androstenedione was measured by chemiluminescence immunoassay (Maglumi 2000 Plus, Snibe, China). Inter-assay coefficients of variation were < 10%. For calculation of the free androgen index (FAI), total testosterone was converted to nmol/L (testosterone[nmol/L] = testosterone[ng/mL] × 3.467), and FAI was computed as (testosterone[nmol/L] / SHBG[nmol/L]) × 100 [20]. Higher FAI indicates greater circulating free androgen relative to binding capacity.

Analyses were performed using SPSS version 17.0. Continuous variables were assessed for normality; group comparisons used an independent samples t-test or a Mann–Whitney U test as appropriate. Categorical variables were compared using chi-square or Fisher’s exact tests. Because the sampling phase differed markedly between groups, sensitivity analyses repeated hormone comparisons after stratification by sampling time (antepartum vs postpartum). Spearman rank correlations were calculated between maternal hormonal markers (total testosterone, estradiol, DHEA-S, androstenedione, SHBG, and FAI) and birth weight percentile and AC percentile at the last ultrasound, separately within the IUGR and control groups. To assess whether the two nominally significant IUGR-group correlations were independent of baseline confounders, exploratory multivariable linear regression models were fitted within the IUGR group with birth weight percentile as the dependent variable and SHBG as the predictor of interest, and with AC percentile as the dependent variable and total testosterone as the predictor of interest; both models were adjusted for maternal age, systolic blood pressure, and sampling time. Correlation and regression analyses were exploratory, complete-case, and no adjustment for multiple comparisons was applied. Two-tailed *p* values < 0.05 were considered statistically significant.

## Results

### Baseline characteristics

**Fig. 1:**
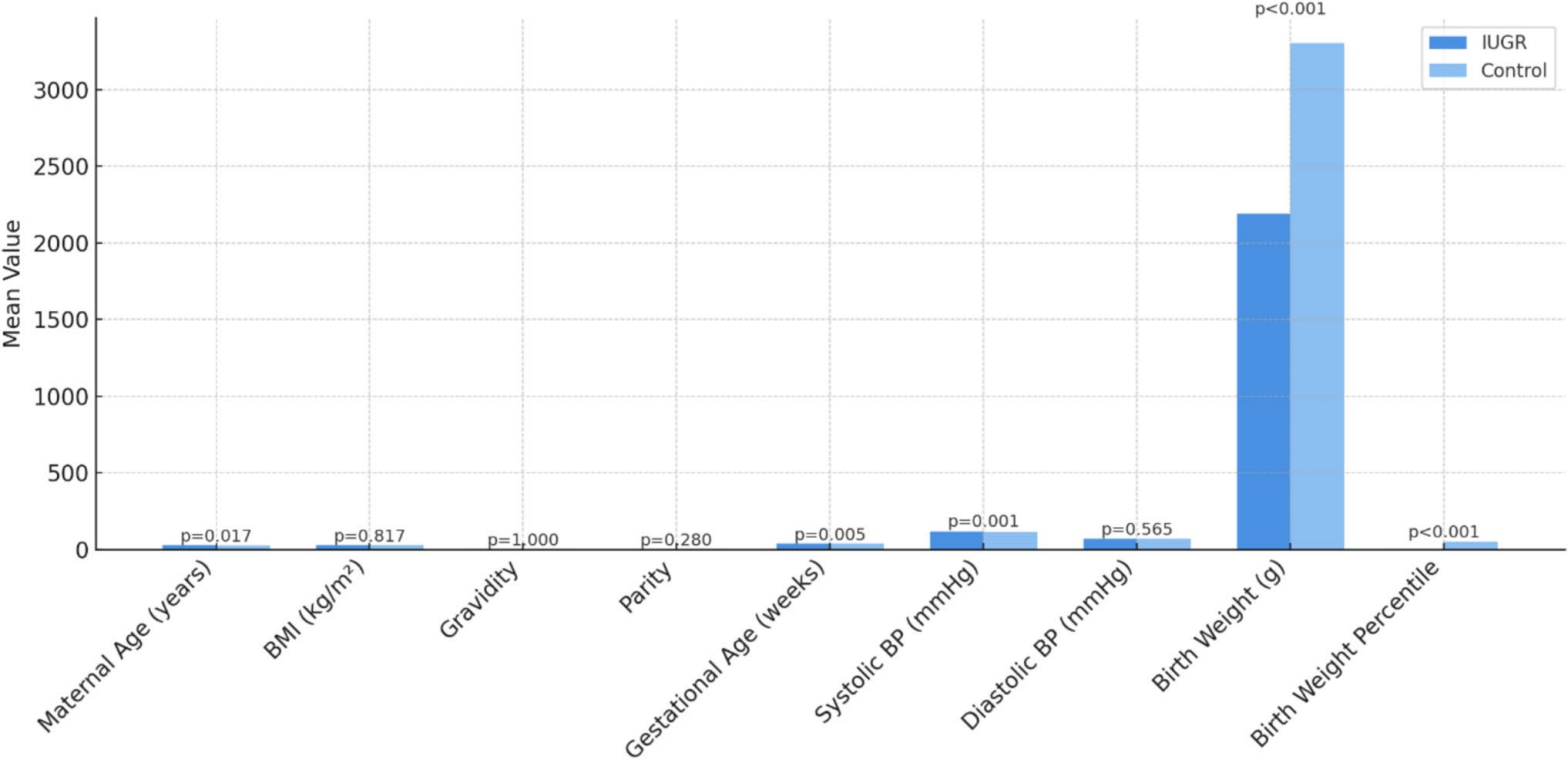
Maternal and fetal characteristics in IUGR and control groups: comparison of mean values for maternal age, body mass index (BMI), gravidity, parity, gestational age at delivery, systolic and diastolic blood pressure, birth weight, and birth weight percentile between intrauterine growth restriction (IUGR) and control groups. Bars represent group means. Asterisks denote statistically significant differences (*p < 0.05, **p < 0.01, ***p < 0.001). Birth weight, birth weight percentile, and gestational age at delivery were lower in the IUGR group, whereas maternal age and systolic blood pressure were higher

A total of 95 women were analyzed (45 IUGR cases and 50 controls). Baseline characteristics are shown in Table 1 (and Fig. 1). Prepregnancy BMI, gravidity, and parity did not differ between groups. Maternal age was higher in the IUGR group than in controls (30.5 ± 6.4 vs 27.2 ± 6.2 years, *p* = 0.017). Gestational age at delivery was lower in IUGR (36.8 ± 3.4 vs 38.6 ± 1.2 weeks, *p* = 0.005), and oligohydramnios occurred in 35.6% of IUGR pregnancies versus 0% of controls (*p* < 0.001). As expected, birth weight and birth weight percentile were lower in IUGR (2190 ± 624 g; ~ 5th percentile) than in controls (3306 ± 391 g; ~ 52nd percentile; both p < 0.001). Fetal sex distribution was similar (male: 40.0% vs 54.0%, *p* = 0.172). Mean systolic blood pressure was modestly higher in IUGR (118.5 ± 7.5 vs 111.8 ± 10.0 mmHg, *p* = 0.001), while diastolic blood pressure did not differ (*p* = 0.565).

**Table 1:** Maternal and fetal baseline characteristics of the IUGR and control groups

Characteristic	IUGR (*n* = 45)	Control (*n* = 50)	*p*-value
Maternal age (years)	30.5 ± 6.4	27.2 ± 6.2	0.017 **(S)**
Body mass index (kg/m^2^)	28.6 ± 5.4	28.5 ± 4.5	0.817 (NS)
Gravidity (number)	3.24 ± 1.93	3.12 ± 1.55	1.000 (NS)
Parity (number)	1.6 ± 1.4	1.7 ± 1.1	0.280 (NS)
Gestational age at delivery (weeks)	36.8 ± 3.4	38.6 ± 1.2	0.005 **(S)**
Systolic blood pressure (mmHg)	118.5 ± 7.5	111.8 ± 10.0	0.001 **(S)**
Diastolic blood pressure (mmHg)	68.1 ± 10.4	69.8 ± 7.3	0.565 (NS)
Oligohydramnios (AFI < 5 cm),* n *(%)	16 (35.6%)	0 (0%)	< 0.001 **(S)**
Male fetal sex,* n* (%)	18 (40.0%)	27 (54.0%)	0.172 (NS)
Birth weight (g)	2190 ± 624	3306 ± 391	< 0.001 **(S)**
Birth weight percentile	5.2 ± 2.3	52.3 ± 26.4	< 0.001 **(S)**

Data are presented as mean ± SD for continuous variables and *n* (%) for categorical variables

*S* significant, *NS* not significant, *AFI* amniotic fluid index

As expected, Table 1 confirms that the IUGR group had substantially lower fetal growth metrics and a higher incidence of oligohydramnios compared to controls, despite similar maternal baseline characteristics in terms of BMI and parity. The IUGR mothers were slightly older on average and had marginally higher systolic blood pressure. The two groups did not differ significantly in fetal sex ratio.

### Maternal SHBG and androgen levels in IUGR vs control

Maternal hormone concentrations are shown in Table 2. SHBG was lower in the IUGR group than in controls (257 ± 117 vs 382 ± 106 nmol/L, *p* < 0.001), and FAI was modestly higher (1.27 ± 1.06 vs 0.85 ± 0.71, *p* = 0.018). Total testosterone, DHEA-S, androstenedione, and estradiol did not differ between groups (all *p* > 0.3). Estradiol showed substantial variability in both groups; heterogeneous sampling timing, including postpartum collection (IUGR: *n* = 9 (20.0%); controls: *n* = 41 (82.0%)), may have contributed to this dispersion.

**Table 2:** Comparison of maternal serum hormonal parameters between IUGR and control pregnancies

Parameter	IUGR (mean ± SD)	Control (mean ± SD)	*p*-value
Total testosterone (ng/mL)	0.85 ± 0.66	0.96 ± 0.81	0.700 (NS)
Estradiol (pg/mL)	2654 ± 5939	3900 ± 7618	0.922 (NS)
DHEA-S (µg/dL)	117.97 ± 90.71	121.34 ± 69.91	0.362 (NS)
Androstenedione (ng/mL)	3.61 ± 2.47	3.62 ± 2.08	0.629 (NS)
**SHBG (nmol/L)**	**257 ± 117**	**382 ± 106**	< 0.001 **(S)**
**Free androgen index (FAI)**	**1.27 ± 1.06**	**0.85 ± 0.71**	0.018 **(S)**

Boldface indicates significant differences (S)

*SHBG* sex hormone–binding globulin, *DHEA-S* dehydroepiandrosterone sulfate, *FAI* (total testosterone[nmol/L] / SHBG [nmol/L]) × 100 after conversion of testosterone from ng/mL to nmol/L using a factor of 3.467. *NS* not significant

As shown in Table 2, maternal SHBG was significantly lower in the IUGR group, whereas FAI was only modestly higher overall. To address the imbalance in the sampling phase, we repeated the analysis within timing strata. In the antepartum subgroup, SHBG remained lower in IUGR (261 ± 128 vs 384 ± 123 nmol/L, *p* = 0.003), whereas FAI did not differ significantly (1.18 ± 1.10 vs 0.85 ± 0.83, *p* = 0.289). In the postpartum subgroup, SHBG also remained lower (240 ± 63 vs 382 ± 104 nmol/L, *p* < 0.001) and FAI was higher (1.61 ± 0.84 vs 0.85 ± 0.69, *p* = 0.010), although this analysis was limited by the small postpartum IUGR sample (*n* = 9). These findings suggest that SHBG is the more reproducible between-group signal in this dataset, whereas the pooled FAI difference is sensitive to sampling phase.

### Correlation of maternal hormones with fetal growth parameters

**Fig. 2:**
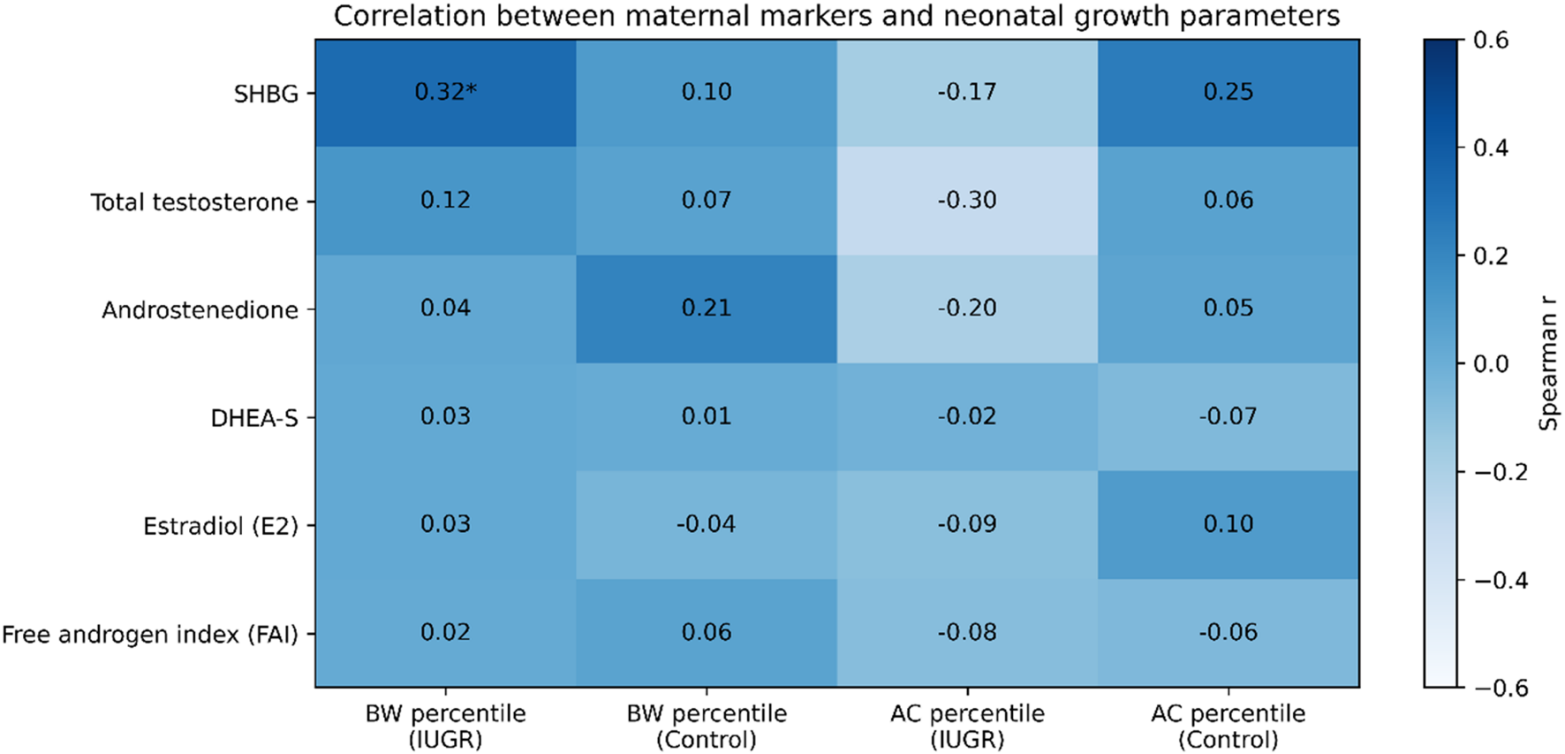
Correlations of maternal hormonal markers with fetal growth parameters in the IUGR group. Scatter plots illustrating the associations between maternal SHBG and birth weight percentile, and between maternal total testosterone and abdominal circumference percentile at the last ultrasound in the IUGR group. Spearman correlation coefficients (*r*) and corresponding p values are shown on the plots

Spearman correlations between maternal hormones and birth weight percentile and abdominal circumference (AC) percentile are presented in Table 3 (Fig. 2). In the IUGR group, maternal SHBG correlated positively with birth weight percentile (*r* = 0.324, *p* = 0.034), and maternal total testosterone showed a borderline inverse correlation with AC percentile (*r* =  −0.298, *p* = 0.050). No other significant correlations were observed in the IUGR group, including FAI (birth weight percentile *p* = 0.885; AC percentile *p* = 0.598), DHEA-S, androstenedione, or estradiol (all *p* > 0.2). In controls, none of the maternal hormones correlated significantly with birth weight or AC percentiles (all *p* > 0.1). To examine potential confounding, we fitted exploratory multivariable linear regression models within the IUGR group adjusting for maternal age, systolic blood pressure, and sampling time. In these models, neither SHBG for birth weight percentile (*β* = 0.004 per nmol/L, *p* = 0.166) nor total testosterone for AC percentile (*β* =  −2.35 per ng/mL, *p* = 0.070) remained statistically significant.

**Table 3:** Spearman correlation coefficients (*r*) between maternal hormonal markers and fetal growth percentiles in IUGR vs. control pregnancies

Maternal marker	IUGR: birth weight percentile (*r*, *p*)	Control: birth weight percentile (*r*, *p*)	IUGR: AC percentile (*r*, *p*)	Control: AC percentile (*r*, *p*)
Total testosterone	0.123 (*p* = 0.427)	0.065 (*p* = 0.660)	–0.298 (*p* = 0.050)	0.064 (*p* = 0.662)
Estradiol (E2)	0.028 (*p* = 0.861)	–0.040 (*p* = 0.786)	–0.090 (*p* = 0.566)	0.097 (*p* = 0.507)
DHEA-S	0.025 (*p* = 0.872)	0.012 (*p* = 0.932)	–0.019 (*p* = 0.903)	–0.065 (*p* = 0.657)
**SHBG**	**0.324 (*p* = 0.034)**	0.099 (*p* = 0.526)	–0.174 (*p* = 0.265)	0.247 (*p* = 0.110)
Androstenedione	0.036 (*p* = 0.824)	0.215 (*p* = 0.166)	–0.197 (*p* = 0.216)	0.047 (*p* = 0.763)
Free androgen index	0.023 (*p* = 0.885)	0.059 (*p* = 0.708)	–0.083 (*p* = 0.598)	–0.057 (*p* = 0.718)

Bold indicates a statistically significant unadjusted correlation (*p* < 0.05) in that group

*AC* abdominal circumference at last ultrasound. *SHBG* sex hormone–binding globulin; *DHEA-S* dehydroepiandrosterone sulfate

As shown in Table 3, in the IUGR group maternal SHBG correlated positively with birth weight percentile (*r* = 0.324, *p* = 0.034), and maternal total testosterone showed a borderline inverse correlation with AC percentile (*r* =  −0.298, *p* = 0.050). These associations were not observed in controls (all *p* > 0.1). However, after adjustment for maternal age, systolic blood pressure, and sampling time, neither association remained independently significant.

## Discussion

In this prospective case–control study, idiopathic IUGR pregnancies showed lower maternal SHBG than controls. FAI was modestly higher in the overall comparison; however, this difference was not maintained in the antepartum-only sensitivity analysis. Within the IUGR group, unadjusted analyses suggested that higher SHBG was associated with a higher birth weight percentile and higher total testosterone with a lower abdominal circumference percentile, but these associations were attenuated after adjustment for maternal age, systolic blood pressure, and sampling time.

### Interpretation of key findings

The markedly lower SHBG observed in mothers of IUGR fetuses is an interesting finding. SHBG is primarily produced by the maternal liver and is upregulated by high estrogen levels. In normal pregnancy, estrogen (especially estriol) from the placenta stimulates a several-fold rise in maternal SHBG [5]. Therefore, a reduction in maternal SHBG in IUGR pregnancies may reflect reduced placental estrogenic drive, altered maternal hepatic regulation, or both. Although we did not detect a statistically significant between-group difference in estradiol, a trend toward lower estradiol in IUGR was observed. The very large standard deviations for estradiol likely reflect heterogeneous sampling relative to delivery, including postpartum collection, as well as marked peripartum hormonal fluctuations. Therefore, the absence of a significant estradiol difference should not be interpreted as evidence of equivalence between groups.

Lower maternal SHBG would be expected to increase the proportion of unbound androgens in the maternal circulation. However, the between-group difference in FAI was modest and was not maintained in the antepartum-only sensitivity analysis. Importantly, total testosterone and DHEA-S levels were not elevated in IUGR mothers compared with controls, suggesting that the most consistent between-group hormonal signal in this dataset is lower SHBG rather than unequivocal androgen excess.

The unadjusted correlations we observed offer preliminary insight. In IUGR pregnancies, higher maternal SHBG was associated with a higher birth weight percentile (i.e., less severe growth restriction), while higher maternal total testosterone was associated with a lower AC percentile. However, after adjustment for maternal age, systolic blood pressure, and sampling time, neither association remained statistically significant. Thus, our data do not establish SHBG or total testosterone as independent correlates of fetal growth severity; rather, these findings should be interpreted as exploratory signals that warrant testing in larger cohorts with standardized antepartum sampling.

Because maternal age, gestational age at delivery, and systolic blood pressure differed between groups, and each of these factors can influence SHBG and steroid concentrations, residual confounding is a plausible alternative explanation for part of the observed associations. In addition, the inclusion of postpartum samples and variable timing relative to ultrasound assessment may have introduced nondifferential measurement error, which would tend to attenuate true relationships. Because postpartum sampling was substantially more frequent in controls than in IUGR cases, between-group comparisons of absolute hormone concentrations, particularly estradiol, may also have been influenced by this sampling imbalance. Furthermore, because multiple exploratory correlation analyses were performed without adjustment for multiple comparisons, some nominally significant or borderline associations may represent type I error and should, therefore, be considered hypothesis-generating.

Although FAI was higher in the pooled between-group comparison, it was not significantly correlated with fetal growth percentiles within the IUGR group, and the antepartum-only subgroup analysis did not show a significant between-group difference. This pattern suggests that the FAI signal is less robust than the SHBG finding in the present dataset. Restricted within-group variability in growth percentiles among cases, the ratio nature of FAI, and sampling heterogeneity may all have contributed to this inconsistency.

Maternal total testosterone in our IUGR group was inversely correlated with fetal AC percentile. AC percentile is a key ultrasound parameter for diagnosing asymmetric IUGR and reflects fetal liver size and subcutaneous fat—essentially a gauge of fetal nutritional status. A negative correlation (r ≈ –0.30) implies that higher maternal testosterone is associated with more severe depletion of fetal abdominal growth. One potential explanation is that when the placenta is compromised, it may have reduced capacity to aromatize androgens into estrogens. In normal pregnancy, much of fetal/maternal DHEA-S is converted by the placenta into estriol; in IUGR, if this conversion falters, androgen levels could accumulate. Elevated maternal androgens might then further impair placental blood flow or directly affect nutrient transfer, exacerbating the growth restriction. This concept finds support in experimental data: injections of testosterone in pregnant rats lead to lower placental and fetal weights and increased placental vascular resistance [8]. Moreover, high maternal testosterone levels have been observed in pregnancies that later develop preeclampsia [21, 22], a condition often overlapping with IUGR due to placental ischemia. Although our study excluded women with overt preeclampsia, the continuum of placental pathology could mean some IUGR cases had mild manifestations of similar vascular insufficiency. It is worth noting that in our control group, maternal hormones did not correlate with fetal size, indicating that within a normal physiological range, variations in maternal SHBG or testosterone have no appreciable impact on a fetus that is growing appropriately. It is the stressed conditions of IUGR that unmask these associations—a classic example of how pathological states can reveal biological relationships that are not apparent in health.

### Comparison with other studies

There is limited literature directly comparable to our findings, as the interplay between maternal sex hormones and IUGR has not been extensively studied. However, our results are consistent with several indirect lines of evidence. Valdés et al. (2012) measured SHBG in early pregnancy and found no significant differences in women who later developed IUGR [18], suggesting (as our data do) that SHBG deviations manifest later, likely as a consequence rather than a cause of growth restriction. A more recent cohort study by Huang et al. (2021) reported sex-dependent associations between maternal androgen levels and offspring weight trajectories from birth to early childhood [23]. This aligns with our observation that increased free androgen (FAI) accompanies lower fetal growth; although we did not find a sex-specific effect (perhaps due to sample size, and our IUGR group actually had more females), the principle that maternal androgen excess correlates with reduced fetal growth is reinforced. Huang’s study also noted complex sex-dependent effects on postnatal catch-up growth [23], highlighting that fetal programming by maternal androgens may have lasting consequences.

Our observation of lower SHBG, and a modest overall increase in FAI, in IUGR mothers resonates to some extent with data from PCOS pregnancies. PCOS is characterized by low SHBG and high FAI; interestingly, in a large Scandinavian cohort, Talmo et al. (2024) reported that infants of mothers with PCOS were modestly smaller, about 130 g on average, than those of non-PCOS mothers, even after adjustment for BMI and other confounders, and they also had lighter placentas on average [7]. The differences in PCOS were not as dramatic as in pathological IUGR, but they do indicate that a hyperandrogenic maternal environment can subtly restrict fetal growth and placental development. In our dataset, however, the FAI difference was attenuated in the antepartum-only sensitivity analysis; therefore, parallels with a clearly hyperandrogenemic state should be drawn cautiously.

Our data suggest that maternal SHBG differs between IUGR and controls near term, whereas evidence for an independent difference in FAI is weaker when sampling phase is considered. Future studies should use standardized antepartum sampling and multivariable models to test whether SHBG or related endocrine markers add value beyond established clinical and ultrasound assessments.

Our findings support the broader literature suggesting that the maternal endocrine milieu differs in pregnancies complicated by intrauterine growth restriction (IUGR), potentially reflecting placental dysfunction and/or maternal metabolic regulation of sex hormone-binding globulin (SHBG). A plausible synthesis is that reduced placental estrogenic signaling lowers maternal SHBG; however, because sampling was heterogeneous (including postpartum samples), conditions were not standardized, and the FAI signal was not robust in the antepartum-only analysis, mechanistic inferences regarding increased free androgen availability should remain tentative. Offspring outcomes were not assessed, so any developmental programming implications remain speculative.

### Clinical implications

The findings from our study, if corroborated by larger research, could have clinical relevance. The most consistent endocrine difference in the present dataset was lower maternal SHBG in IUGR pregnancies. SHBG is a relatively stable protein that can be measured with routine immunoassays. While early-pregnancy SHBG screening did not prove predictive in prior studies [19], its utility may lie later in gestation: an inappropriately low sex hormone-binding globulin (SHBG) in mid-gestation or the third trimester (relative to gestational norms) could signal evolving placental insufficiency or intrauterine growth restriction (IUGR), analogous to low placental growth factor (PlGF) in late pregnancy. In our data, SHBG was already reduced once IUGR was clinically evident; prospective studies should test whether SHBG declines before ultrasound diagnosis.

Mechanistically, future studies should combine maternal metabolic profiling (e.g., insulin resistance markers) with placental functional assessments to determine whether low SHBG reflects reduced placental estrogenic drive, maternal metabolic regulation of hepatic SHBG production, or both. Longitudinal sampling across gestation is also needed to clarify whether changes in SHBG and FAI precede, coincide with, or follow fetal growth restriction.

Pathophysiologically, our findings may indicate that IUGR is accompanied by altered maternal sex hormone-binding rather than clearly demonstrable independent free-androgen excess. This underscores the mother–placenta–fetus interdependence: placental dysfunction may be mirrored by measurable maternal changes (including liver-derived SHBG), which may in turn influence the intrauterine milieu. However, stronger mechanistic inferences will require standardized antepartum sampling and larger adjusted analyses.

### Strengths and limitations

This study’s strengths include its prospective design and a well-defined IUGR cohort excluding major confounders such as pre-eclampsia and major anomalies. We measured a comprehensive sex-steroid and SHBG panel and calculated FAI using standard unit conversion. To our knowledge, few studies have specifically evaluated maternal SHBG and FAI in IUGR, and our timing-stratified sensitivity analyses and adjusted regression models provide a more rigorous assessment of these signals.

Blood sampling was not fully standardized: some participants were sampled within 24 h postpartum, and postpartum sampling was substantially more common in controls (41/50, 82.0%) than in IUGR (9/45, 20.0%), which may have biased absolute concentrations given rapid hormonal changes after placental delivery and may have contributed to estradiol dispersion. Time of day and fasting status were not standardized or consistently recorded. Several maternal and pregnancy characteristics differed between groups (e.g., maternal age, gestational age at delivery, systolic blood pressure). Although we addressed key concerns through timing-stratified sensitivity analyses and multivariable adjustment, residual confounding cannot be excluded. The moderate sample size limited subgroup and multivariable analyses, particularly the antepartum control subgroup and postpartum IUGR subgroup, and the observational design precludes causal inference; placental/fetal functional data (e.g., placental weight/histopathology, fetal adrenal output) were not available. Although overt pre-eclampsia was excluded, subclinical overlap cannot be entirely ruled out.

Despite these limitations, the findings provide a foundation for larger, multi-center prospective studies with standardized antepartum sampling and integrated placental functional readouts (e.g., aromatase activity and other placental markers). Therapeutic implications remain speculative and are beyond the scope of the present observational data.

## Conclusion

In this prospective case–control study, mothers carrying growth-restricted fetuses had lower serum SHBG than mothers with appropriately grown fetuses. FAI was modestly higher overall but was not significantly different in the antepartum-only sensitivity analysis. Within the IUGR group, unadjusted associations between SHBG, total testosterone, and fetal growth parameters did not remain statistically significant after adjustment for maternal age, systolic blood pressure, and sampling time. Accordingly, these findings should be interpreted as hypothesis-generating rather than evidence of clinical utility or causality. Larger prospective cohorts with standardized antepartum sampling, gestation-specific reference ranges, and confounder-adjusted models are needed to confirm these associations and to determine whether any biomarker signal adds incremental value beyond established clinical and ultrasound assessment.

## Data Availability

The datasets generated and/or analyzed during the current study are not publicly available due to institutional and ethical restrictions but are available from the corresponding author on reasonable request.
